# The emerging roles of CDK12 in tumorigenesis

**DOI:** 10.1186/s13008-017-0033-x

**Published:** 2017-10-27

**Authors:** Hana Paculová, Jiří Kohoutek

**Affiliations:** Department of Chemistry and Toxicology, Veterinary Research Institute, Hudcova 296/70, Brno, 621 00 Czech Republic

**Keywords:** CDK12, RNA pol II, Suppressor, Oncogene, Dinaciclib, THZ531

## Abstract

Cyclin-dependent kinases (CDKs) are key regulators of both cell cycle progression and transcription. Since dysregulation of CDKs is a frequently occurring event driving tumorigenesis, CDKs have been tested extensively as targets for cancer therapy. Cyclin-dependent kinase 12 (CDK12) is a transcription-associated kinase which participates in various cellular processes, including DNA damage response, development and cellular differentiation, as well as splicing and pre-mRNA processing. CDK12 mutations and amplification have been recently reported in different types of malignancies, including loss-of-function mutations in high-grade serous ovarian carcinomas, and that has led to assumption that CDK12 is a tumor suppressor. On the contrary, CDK12 overexpression in other tumors suggests the possibility that CDK12 has oncogenic properties, similarly to other transcription-associated kinases. In this review, we discuss current knowledge concerning the role of CDK12 in ovarian and breast tumorigenesis and the potential for chemical inhibitors of CDK12 in future cancer treatment.

## Background

Cyclin-dependent kinases (CDKs) are principal regulators of various cellular processes. They are divided into two subfamilies: cell cycle-associated CDKs (CDK1, 2, 4, 6), which directly regulate progression through individual cell cycle phases, and transcription-associated CDKs (CDK7, 8, 9, 11, 12, 13), which regulate gene transcription. These kinases phosphorylate the C-terminal domain (CTD) of Rbp1, the largest subunit of RNA polymerase II (RNA pol II) as well as various transcription regulatory factors. Since CDKs are frequently dysregulated in tumor cells, therefore they are attractive therapeutic targets for a broad spectrum of tumors [1, 2].

Eukaryotic transcription is very complex and tightly regulated. Essential cellular processes, including differentiation and response to extracellular stimuli, depend on regulation at the transcriptional level [3]. In addition, precise coordination of transcription with other events, such as mRNA processing, splicing, chromatin remodeling, and modification of histones is crucial for normal cellular physiology. Consequently, deregulation of these processes drives cancer onset and progression [4].

Transcription factors are frequently mutated in cancer cells and represent typical oncogenes and tumor suppressors. These mutations lead to alterations in the gene expression programs and might create dependency on certain transcriptional regulators making cancer cells addicted to their activities [5]. Such a phenomenon is called “Transcriptional Addiction”, and it provides opportunities for novel therapeutic interventions in cancer [5].

CDK12 is a transcription-associated CDK that phosphorylates the CTD of RNA pol II and it is essential for DNA damage response (DDR), splicing, and differentiation [6]. CDK12 mutations as well as overexpression have been reported in various malignancies. Subsequently, two CDK12 inhibitors were developed independently. They will be instrumental for studying physiological function of CDK12 and could be tested as anti-cancer drugs [7, 8]. In this review, we summarize current knowledge concerning CDK12 role in tumorigenesis and its therapeutic potential.

## CDKs in transcription regulation

Transcription is a complex process coordinated by numerous factors. Posttranslational modifications (particularly phosphorylation) of RNA pol II CTD constitute one of the crucial mechanisms of transcription regulation [3, 4]. RNA pol II is a multi-subunit complex responsible for RNA synthesis of eukaryotic genes coding most proteins and small RNAs. Modifications of CTD, which consists of repeated YSPTSPS heptapeptides, form patterns that enable specific binding of various factors coordinating transcription as well as co-transcriptional pre-mRNA processing [3, 4]. Phosphorylation of serines, threonine and tyrosine, among other post-translational modifications, is essential for the transition between individual phases of transcription, such as initiation, elongation, termination, and individual steps of pre-mRNA processing. The majority of kinases phosphorylating CTD of RNA pol II belong to the group of transcription-related CDKs. CDK7 is a part of transcription initiation factor TFIIH. CDK9, responsible for the activity of P-TEFb, is associated with early elongation [3, 4].

## CDK12 structure, homologs and associating cyclin

In 2001, a new transcription-related kinase was discovered. It was described as a Cdc2-related kinase with an arginine/serine-rich (RS) domain (CrkRS). The CrkRS consists of 1490 amino acids and has a kinase domain, proline-rich regions and a serine-rich domain, which is typical for splicing factors from the SR protein family [9].

Later on, cyclins L1 and L2 were described as CDK12-associating cyclins and the CrkRS was renamed to CDK12 [10]. Nevertheless, the cloning of *Drosophila* CDK12 led to identification of cyclin K as a *bona fide* CDK12-associating cyclin. The cyclin K/CDK12 complex was demonstrated to phosphorylate the CTD of RNA pol II in vitro, and CDK12 was established as a CTD kinase [11]. The association between cyclin K and CDK12 was further confirmed by mass spectrometry and immunoprecipitation in mammalian cells [12, 13]. Additionally, the ability of CDK12 to phosphorylate the CTD of RNA pol II was clearly demonstrated [12]. Analogically to CDK9, CDK12 is expected to phosphorylate additional substrates other than CTD, such as transcription or splicing factors, which may be critical for CDK12 role in regulation of transcription and related processes. Additional CDK12 phosphorylation targets and related biological functions remain to be determined. The crystal structure of CDK12 was later described, opening new possibilities to study its function and its potential as a drug target and to develop specific CDK12 inhibitors [14]. The closest CDK12 human homologue is CDK13 (also known as CDC2L5, CHED). It contains a kinase domain which displays high sequence identity to CDK12 kinase domain, but its sequence is unrelated on C- and N- terminus. CDK13 associates with Cyclin K and forms a separate complex [12]. Similarly to CDK12, CDK13 is capable of phosphorylating CTD [12]. Nevertheless, CDK13 is much less studied than CDK12 and its function is less clear. Due to the sequence similarity, one can expect a redundancy or overlap in functions of these two kinases.

In yeast (*S. cerevisiae*), there are two kinases capable of phosphorylating Ser2 of CTD, Ctk1 and Bur1. Before CDK12 and CDK13 were discovered, it was assumed that CDK9 is the only metazoan orthologue of Ctk1 and Bur1. Based on evolutionary and functional evidence Bartkowiak et al. identified *Drosophila* CDK12 and human CDK12 and CDK13 as an ortholog of yeast Ctk1, while Bur1 is CDK9 orthologue [11, 15] Lsk1, less studied Ser2 CTD phosphorylating protein, is CDK12 ortholog in *S. pombe* [11].

## CDK12 in transcription regulation

Similarly to CDK9, CDK12 is associated with transcription elongation and is able to phosphorylate the RNA pol II CTD serine at position 2 (Ser2) in *Drosophila* [11] and in human cells [12, 13]. However, downregulation of CDK12 activity does not affect the global transcription rate, and when CDK12 is depleted from cells, transcription of a unique subset of genes is altered (interestingly mainly the genes necessary for DDR). Depletion of CDK12 and Cyclin K results in decreased expression of long genes (> 10 kb) and genes with higher number of exons. Observations have led to the hypothesis that CDK12 is a kinase that promotes transcription of a set of specific genes [12]. Supporting this hypothesis, CDK12 was found to be necessary for the expression of Nrf2-dependent genes in *Drosophila* cells, where CDK12 does not affect the overall transcription but is rather involved in the transcriptional stress response [16].

Nevertheless, recent studies provide arguments against a hypothesis that CDK12 is a gene-specific CDK kinase. According to this concept, CDK12 is actively recruited to the body of transcribed genes by fully operated Pol II associated factor 1 (Paf1) right after paused RNA pol II is released into the productive elongation phase [17]. Also, when specific anti-CDK12 antibody was used in a ChIP-Seq experiment, it was found that CDK12 binds to promoters and bodies of protein-coding genes and to active transcription enhancers, with the ChIP-seq signal overlapping with the RNA pol II signal [8]. The same group developed a specific CDK12 inhibitor and identified CDK12-responsive genes in microarray experiments. Critically, a lower dose of the CDK12 inhibitor reduced transcription of core DDR genes (including BRCA1, FANCF and ERCC4) and higher inhibitor dose decreased expression of super-enhancer-associated genes compared to genes associated with typical enhancers [8].

An additional aspect related to CDK12 function in transcription is the specificity of CDK12 in relation to the specific serine within CTD. In the classical view, CDK12 phosphorylates CTD Ser2 in vivo and in vitro. In contrast to CDK9, CDK12 is responsible for Ser2 phosphorylation on the 3′ prime end of genes [8]. In an attempt to clarify CDK12 substrate preference, the Geyer group performed in vitro kinase assays and immunoprecipitation experiments. They described CDK12 ability to phosphorylate both CTD Ser2 and Ser5; however, it needs pre-phosphorylation of CTD on Ser7 for optimal activity [14]. Nonetheless, inhibition of CDK12 in HeLa cells led to a severe block of cell growth but global phosphorylation of individual CTD related serines was affected only moderately [18]. Finally, CDK12 inhibition leads to a reduction in global Ser2 but not Ser5 or Ser7 phosphorylation of the CTD [8]. To date, CTD substrate specificity studies have been dependent on antibodies against specific CTD modifications, such as H5, H14, and antibodies prepared by the Eick group [19]. The specificity of these antibodies seems to be affected by neighboring modifications [18], which could bias the results and make it challenging to draw conclusions. A cellular system suitable for mass spectroscopy analyses of CTD of RNA pol II modifications was recently developed. It provides a promising tool for elucidating the specificity of CDK12 and other CDKs in relation to individual CTD serines [20].

In addition to regulating the elongation phase of transcription, CDK12 participates in transcription termination. Polyadenylation-coupled phosphorylation of Ser2 at the 3′ end of the *MYC* gene by CDK12 is necessary for recruitment of polyadenylation factor CstF77 and is therefore necessary for effective transcription termination [21]. Similarly, CDK12 depletion leads to reduced Ser2 phosphorylation and cleavage stimulation factor 64 (CstF64), thereby leading to impaired 3′ end processing of the *c*-*FOS* gene after activation of EGF signaling [22]. These two studies clearly demonstrated how regulation of transcription is coupled to pre-mRNA processing by recruiting different factors to modified CTD.

It is evident that transcription elongation and termination serve as regulatory steps in gene expression. Dysregulation of these processes may alter levels of tumor suppressors or oncogenes and possibly result in tumorigenesis. Nevertheless, the exact role of CDK12 in transcription regulation is not fully understood. The fundamental question remains unanswered as to whether CDK12 affects transcription globally or if it is kinase-specific for a unique set of genes, such as DDR or super-enhancer-associated genes? Similarly to CDK9, CDK12 could bind and phosphorylate also additional factors and thus regulate transcription employing CTD phosphorylation independent mechanisms.

## CDK12 function in splicing

Since its discovery, it has been known that CDK12 co–localizes with SC35 (also known as SRSF2 or SFRS2), a spliceosome component, and contains the RS domain which is typical for RNA-interacting and splicing factors [9]. Supporting CDK12 involvement in RNA-splicing machinery, three studies independently identified several factors of the splicing apparatus and components of nuclear speckles to be putative CDK12-associating partners based on mass spectrometry analyses [18, 22, 23]. Nevertheless, most of these associations have not yet been confirmed by immunoprecipitation with endogenous CDK12. One example of CDK12 involvement in splicing is based on the observation that depletion of CDK12 leads to dysregulated alternative splicing of serine/arginine splicing factor1 (SRSF1). In addition to its association with splicing factors, CDK12 was demonstrated to co-immunoprecipitate proteins of exon junction complexes and RNA-binding proteins [22]. In addition, *Drosophila* CDK12 is involved in the alternative splicing of *neurexin IV* in coordination with mRNA–binding protein *HOW* during *Drosophila* nervous system development [24].

A recent study confirmed that CDK12 is involved in splicing. The authors described interaction of CDK12 with spliceosome components and splicing regulatory factors using immunoprecipitation followed by mass spectroscopy [25]. In RNA-seq experiments, Tien et al. described a new role of CDK12 in splicing: CDK12 regulates alternative last exon splicing, gene- and cell type-specific specialized type of alternative splicing. In breast cancer cells, depletion or overexpression of CDK12 leads to altered alternative last exon splicing of a subset of genes and may contribute to tumorigenesis [25].

Despite a growing body of evidence supporting CDK12 involvement in splicing, the precise role of CDK12 in this process as well as other co-transcriptional events is yet to be elucidated. Description of additional binding factors and new potential phosphorylation substrates may clarify the precise function of CDK12 in this process. CDK12 could form a functional link between transcription regulation and co-transcriptional pre-mRNA splicing. Alternative splicing affects a large number of transcripts in mammals and provides regulation for the majority of cellular processes. Aberrant splicing of various regulatory factors also leads to tumorigenesis [26–28] providing one of the explanatory roles for CDK12 deficiency in tumorigenesis via splicing dysregulation.

## CDK12 in development

Even though CDK12 is ubiquitously expressed, the CDK12 protein level differs in particular tissues. High human CDK12 levels can be found in testes, ovaries, leukocytes and adrenal gland, measured by mRNA levels [29]. High mouse CDK12 protein levels are in testes as well as in highly proliferative tissues and mouse embryonic stem cells [30]. This suggests that CDK12 could have tissue-specific roles in cellular commitment and differentiation. Several studies have pointed out CDK12 function in neuronal development and differentiation [31–33]. For instance, depletion of CDK12 (and CDK13) leads to reduced axonal outgrowth mediated probably by lowered CDK5 expression [32]. Ser2 phosphorylation in *C. elegans* germline depends on the activity of CDK12/cyclin K rather than on CDK9 [34].

As is evident from mouse studies, both CDK12 and its associating cyclin K are essential for early embryogenesis in mice. In vitro cultured CDK12 −/− blastocysts fail to undergo inner cell mass outgrowth due to increased apoptosis and impaired repair of DNA damage [33].

CDK12 associating cyclin K is highly expressed in murine embryonic stem cells but not in their differentiated derivatives. The cyclin K protein level decreases with differentiation and correlates with levels of Oct4, Sox and Nanog proteins known to be necessary for maintaining stemness. These observations suggest that cyclin K/CDK12 and also cyclin K/CDK13 complexes take part in maintaining the self-renewal capacity of murine embryonic stem cells [35].

One of the features of various tumors is stemness and cell dedifferentiation. Since CDK12 maintains a dedifferentiated state in mouse embryonic stem cells, one could envision a scenario where CDK12 maintains the dedifferentiated state of cancer stem cells. High CDK12 activity would therefore accelerate tumor progression and therapy resistance, paradoxically to its proposed role as a tumor suppressor.

## CDK12 role in DNA damage response

Even though the exact function of CDK12 is not fully understood, it is evident that it plays a significant role in DDR by affecting the expression of genes involved in homologous recombination (HR) promoted DNA damage repair and probably also other repair pathways [7, 12, 36]. Consequently, CDK12 silencing results in increased endogenous DNA damage [12]. Cells expressing catalytically inactive mutant forms of CDK12 exhibit a compromised ability to effectively execute HR [36]. CDK12 −/− cells derived from mouse blastocysts show decreased expression of DDR genes and increased levels of DNA damage [33]. Administration of CDK12 inhibitor THZ531 also reduces expression of DDR-associated genes [8].

However, a recent study pointed out that the DNA damage pattern in ovarian tumors with CDK12 loss is different than in the case of loss of HR-associated genes. Samples bearing inactive CDK12 have been shown to contain large tandem duplications rather than markers of impaired HR [37].

Cyclin K (in addition to BRCA1) was found in a global screen for genes sensitizing cells to the DNA-damaging drug camptothecin [38]. Furthermore, downregulation of CDK12 leads not only to spontaneous cell death but also to sensitization of cells to various DNA-damaging agents such as etoposide, mitomycin C and camptothecin [12]. Also, mutant, inactive forms of CDK12 sensitize cancer cells to cisplatin [39]. CDK12 deficiency sensitizes cells to inhibitors of PARP1/2, an important factor involved in DNA repair (discussed further in more detail) [7, 39, 40].

Despite the fact that CDK12 role in DDR is not yet fully understood, it is clear that CDK12 is necessary for maintaining genomic stability and functional DDR, particularly HR promoted DNA damage repair. Impaired DDR and accumulation of DNA damage is a typical hallmark of cancer [41], which directly links CDK12 deficiency to tumorigenesis.

## Transcriptional CDKs and cancer

CDKs are principal regulators of the cell cycle and consequently participate in control of cell proliferation. Each CDK plays a distinct role in this process and is activated in coordination with multiple factors. Dysregulation of CDKs is a feature typical of a large number of tumor types. Hence, CDKs are attractive targets for cancer therapy and numerous CDK inhibitors have been synthesized and tested as anti-cancer drugs [1, 42]. In addition to cell cycle-associated CDKs, transcription-associated CDKs have emerged as prospective therapeutic targets, exploiting the so-called transcriptional addiction, According to this concept, cancer cells depend on dysregulated transcriptional programs maintained by principal transcriptional regulators, among them transcription-associated CDKs [5]. An increasing number of studies have pointed out the connection between individual transcriptional CDKs and cancerogenesis [5].

CDK9, a kinase responsible for the activity of positive transcription elongation factor (P-TEFb), regulates transition from the initiation to the productive elongation phase of transcription. Overexpression of oncogenic transcription factor c-Myc leads to increased activity of CDK9 and enhances the current transcriptional program by stimulating RNA pol II elongation [43]. Consequently, CDK9 inhibitors have been shown to limit proliferation in Myc-overexpressing liver cancer cells [44] and B cell lymphoma [45]. CDK9 is activated in different types of leukemia. MLL, a histone methyltransferase, frequently fuses with components of the super-elongation complex to form oncogenic factors which activate P-TEFb and promote transcription. Inhibition of CDK9 subsequently limits proliferation of these cells [46].

After CDK13 amplification was described in hepatocellular cancer, CDK13 was consequently proposed to be an oncogene [47]. A high level of CDK11 in breast cancer correlates with clinicopathological parameters. CDK11 downregulation limits cell proliferation and migration in breast cancer cell lines. Targeting CDK11 has been proposed for breast cancer treatment [48].

In conclusion, overstimulation of transcription-associated CDKs promotes proliferation of various cancer types. Analogously, cancer cells may depend on CDK12 and thus it can serve as a therapeutic target. Importantly, CDK12 overexpression has been documented in breast tumors [49, 50].

## CDK12 inhibitors

The anti-tumor potential of various CDK inhibitors has been tested in clinical trials. In addition to the pan-selective inhibitors such as flavopiridol and roscovitine, inhibitors showing specificity for individual CDKs have been developed targeting cell cycle- as well as transcription-linked CDKs [1]. Taking into account that CDK12 plays a critical role in multiple cellular processes and is mutated or overexpressed in various types of cancer, CDK12 inhibition emerges as a favorable strategy for cancer treatment. Two studies recently described CDK12 inhibitors with different chemical structure and specificity range.

Initially, dinaciclib (SCH 727965) was described as a potent inhibitor of CDK2, CDK5, CDK1 and CDK9 exhibiting an anti-proliferative effect in various cell lines [51]. Johnson et al. (2016) discovered that dinaciclib potently inhibits also CDK12 with IC_50_ comparable to CDK9. Dinaciclib administration, similarly to CDK12 silencing, leads to reduced expression of HR genes and reduced RNA pol II CTD Ser2 phosphorylation, and the effects of dinaciclib are thus reminiscent of CDK12 inhibition. Moreover, BRCA1 wild-type cells treated with dinaciclib exhibit compromised HR, which conveys a sensitivity to PARP1 inhibitors. Importantly, combined treatment with PARP1/2 inhibitor veliparib and CDK12 inhibitor dinaciclib efficiently inhibited tumor growth in a patient-derived xenograft model. These findings foresee a possibility to use a CDK12 inhibitor to sensitize or reverse PARP1/2 inhibitor resistance in tumors [7].

CDK7 inhibitor THZ1 has recently been shown to limit the transcription of factors dependent on super-enhancers, among them MYC proto-oncogenes, and it has been pre-clinically tested for treatment of lung carcinoma [43], T-cell acute lymphoblastic leukemia [44], and triple negative breast cancer [45]. THZ1 inhibits CDK12 at higher concentrations and its biological effect could be partly ascribed to CDK12 inhibition [52].

A selective and potent CDK12/13 inhibitor TZH531 was recently developed [8]. The attempt to synthesize a CDK12 inhibitor was based on THZ1, which covalently binds cysteine 312 located on an extension of the CDK7 kinase domain. CDK12 and CDK13 possess cysteines 1039/1017 in a similar extension close to the kinase domain. The authors exploited structural differences between CDK7 Cys312 and CDK12/13 Cys 1039/1017 and screened for an inhibitor specific solely for CDK12/13. THZ531 selectively inhibits CDK12/13 activity 50 times more efficiently than CDK7 or CDK9. In cells, THZ531 induced apoptosis, inhibited elongation of genes and led to reduced expression of DDR and super-enhancer dependent genes. THZ531 exhibited an antiproliferative effect in Jurkat T-cell acute lymphoblastic leukemia cells [8]. Yet, respective contributions of CDK12 and CDK13 to observed biological effects are not known [40].

In summary, CDK12 inhibitors show promise as anti-cancer drugs, either as a stand-alone treatment or in combination with other compounds such as PARP1/2 inhibitors.

## CDK12 mutations in high-grade serous ovarian cancer (HGSOC)

Genomic instability is a typical feature of various malignancies. Mutations in DDR genes resulting in accumulation of DNA damage are often driving progressive events in cancerogenesis. Defective DNA repair machinery results in accumulation of mutations and accelerated cancer transformation and progression [41].

HR deficiency and genomic instability are characteristic for about 50% of HGSOC [53]. HR-associated genes such as BRCA1 or BRCA2 are mutated most frequently and represent typical tumor suppressors. In addition to p53, BRCA1 and BRCA2, CDK12 is one of only nine recurrently mutated genes; it is mutated in about 3% of HGSOC cases [53]. Additional studies have described these mutations in more detail. Mostly they are homozygous point mutations in the CDK12 kinase domain leading to the loss of CDK12 function [36]. Mutant CDK12 forms have compromised ability to phosphorylate RNA pol II CTD, and cells display impairment in HR promoted DNA repair. This is caused predominantly by an inability to bind cyclin K [36, 39]. Furthermore, two studies have pointed out that in patients samples, BRCA1, BRCA2 and CDK12 mutations were mutually exclusive [40, 54]. This observation strongly suggests that BRCA1 and CDK12 participate in one regulatory pathway and supports the hypothesis that CDK12 controls expression of BRCA1 and other DDR genes. This suggestion can be further supported by the fact that more of the key DDR proteins were observed to be deregulated in patient tumor samples bearing CDK12 mutations [36]. Considering these observations, CDK12 was suggested to be a tumor suppressor.

## CDK12 loss confers sensitivity to PARP1/2 inhibitors

Despite its contribution to tumor promotion, genomic instability also provides an opportunity for cancer therapy. Inhibitors of PARP1, a protein which participates in DDR, require defective HR for their anti-cancer activity. As defective HR is common in some tumors, PARP1 inhibition is becoming synthetically lethal to such cells [41]. Hence, loss-of-function mutations of HR regulators BRCA1 and BRCA2 are markers of application of PARP1/2 inhibitors-based therapy [55, 56]. Although PARP1/2 inhibitors have recently been translated into clinics, certain tumors develop resistance to PARP1/2 inhibitors, and new strategies for restoring PARP1/2 inhibitors sensitivity are needed [57].

A synthetic lethality screen determined *CDK12* to be one of the additional genes conferring sensitivity to the PARP1/2 inhibitor olaparib [40]. Ovarian cancer cell lines with lower expression of CDK12 are more sensitive to olaparib treatment, and downregulation of CDK12 leads to increased olaparib sensitivity. The therapeutic effect of olaparib on CDK12-silenced tumor cells was confirmed in vivo in xenograft experiments [40]. Increased sensitivity of CDK12-compromised cells to cisplatin, the alkylating agent melphalan, and the PARP1 inhibitor veliparib was observed in a CDK12-silenced ovarian cancer cell line [39]. In addition, Her2-positive breast cancer cells with downregulated CDK12 display sensitivity to PARP1/2 inhibitors [58]. Finally, the CDK12 inhibitor dinaciclib in combination with the PARP1/2 inhibitor veliparib resulted in inhibition of tumor growth in vitro, in vivo and in a patient-derived xenograft model [7].

Consistent with the fact that CDK12 is necessary for expression of HR genes, loss of CDK12 confers sensitivity to PARP1/2 inhibitors. CDK12 loss in a tumor could serve as another marker for treatment with PARP1/2 inhibitors or additional inhibitors of DDR network, as well as with other DNA-damaging compounds.

## CDK12 dysregulation in breast cancer

In addition to HGSOC, several studies have shown dysregulation of CDK12 in individual subtypes of breast cancer.

Triple-negative breast cancer (TNBC) samples contain mutational spectrum similar to ovarian cancer. These tumors do not amplify any characteristic receptor (ER, HER2, or PR) but display mutations in DDR genes that promote genomic instability. Recurrently mutated genes include p53 (80% of cases) and BRCA1 (30% of cases) [59]. CDK12 mutations were identified in 1.5% of TNBC cases [59, 60]. TNBC patients with defective HR (including loss-of-function mutated CDK12) may benefit from treatment using PARP1/2 inhibitors [59].

A large number of breast tumors are dependent on an overexpressed estrogen receptor (ER) and therefore its targeted inhibition is used for counteracting the tumor growth [61]. CDK12 silencing modifies the sensitivity of ER-positive cells to tamoxifen, a drug blocking ER signaling. CDK12 downregulation activates the mitogen-activated protein kinase (MAPK) pathway, which in turn leads to loss of ER dependency and causes resistance to tamoxifen [62].

Another subtype of breast cancer is characterized by amplification of oncogene HER2 (also known as ERBB2 or EGFR2), a tyrosine kinase receptor, which stimulates cell proliferation and inhibits apoptosis [59]. In breast cancer, HER2 is a part of the frequently amplified and overexpressed 17q12-q21 locus [63]. In addition to HER2, 17q12-q21 amplicon commonly contains several neighboring genes including *MED1*, *GRB7*, *MSL1*, *CASC3* and *TOP2A* [50, 59]. Interestingly, the HER2 amplicon also contains the *CDK12* gene in 71% of cases [50, 64].

HER2-amplified tumors may benefit from therapy based on usage of antibodies against HER2 receptor (trastuzumab, pertuzumab) or tyrosine-kinase inhibitors (lapatinib) [59]. In addition to HER2, overexpression of co-amplified genes might also have an impact on breast cancer development. Additional genes involved in the 17q12-q21 amplicon might therefore be oncogene candidates [63]. Moreover, amplification of additional genes in the 17q12-q21 locus might be responsible for resistance of certain tumors to HER2-targeted therapy. These oncogenic factors, including CDK12, represent potential druggable targets [64]. Mertins and colleagues analyzed the proteome of breast cancer samples and found CDK12 amplification on mRNA and protein level as well as increased CDK12 phosphorylation in HER2-amplified tumors [50]. These observations indicate elevated CDK12 activity in these tumors, and CDK12 has been proposed as an additional druggable target in HER2-amplified breast tumors [50]. In parallel, the diFiore group performed a broad screen of serine/threonine kinases with altered expression in several human cancers, among them breast cancer. They found that CKD12 is upregulated in HER2–positive breast cancer samples and demonstrated a strong correlation between CDK12 level and high tumor grade. They also proposed that a high CDK12 level could serve as a prognostic marker [49].

In 13% of cases, rearrangements in 17q12-q21 amplicons lead to disruption of the CDK12 gene and resulted in CDK12 loss of function and PARP1/2 inhibitor sensitivity of these cells. This observation suggests that the subset of HER2-amplified patients with disrupted CDK12 could benefit from PARP1/2 inhibitor treatment [58]. Additionally, a fusion form of the likely nonfunctional CDK12 gene was also found in a micropapillary breast cancer sample [58].

An increasing number of studies describe loss of function or amplification of CDK12 in breast cancer samples. Loss of function in CDK12 may lead to genomic instability and be predictive of PARP1/2 inhibitor treatment. Tumors displaying CDK12 amplification, on the other hand, may be dependent on its overexpression and CDK12 may provide a new therapeutic target for breast malignancies.

## Is CDK12 a tumor suppressor or does it have oncogenic properties?

Loss of tumor suppressors and addiction to oncogenes are mechanisms driving cancer onset and progression. A growing number of studies describe mutations or amplification of the *CDK12* gene in tumor samples. These data may seem contradictory, since CDK12 functions either as a tumor suppressor or it has features that resemble an oncogene.

In HGSOC and TNBC, CDK12 is a tumor suppressor. CDK12 is necessary for expression of DDR genes and it is essential for HR mediated DNA repair [12, 50]. Consequently, CDK12 loss leads to increased genomic instability, which is a typical feature of these tumors and represents one of the hallmarks of cancer progression [65]. CDK12 loss-of-function mutations or inhibition confers sensitivity of cells to PARP1/2 inhibitors [7, 39, 40].

In a different context, CDK12 has properties that resemble oncogenes. CDK12 amplification, resulting in its overexpression, correlates with more aggressive tumor progression in HER2-positive breast cancers [49]. CDK12 is highly active in these tumors, and it has been proposed as a druggable target in HER2-amplified breast cancer [50]. This idea might be supported by the fact that CDK12 inhibition limits the growth of cancer cells [8, 66]. The concept of transcriptional addiction describes dependency of cancer cells on a certain transcriptional regulator, which maintains an altered transcriptional program [5]. In line with this concept, inhibitors of CDK12-related transcriptional kinases CDK7 and CDK9 reduce proliferation of cancer cells and are being tested as anti-tumor drugs. Similarly to CDK7, CDK12 inhibitors limit the expression of super-enhancer-associated oncogenic transcriptional factors [8, 67]. This suggests that certain tumors might be transcriptionally addicted to CDK12 and so CDK12 inhibition might be a promising anti-cancer strategy. A recent study described that CDK12 overexpression affects alternative last exon splicing. Therefore CDK12 overexpression can increase the invasiveness of a breast cancer cell line, by decreasing the expression of the long isoform of DNAJB6 [25].

**Fig. 1:**
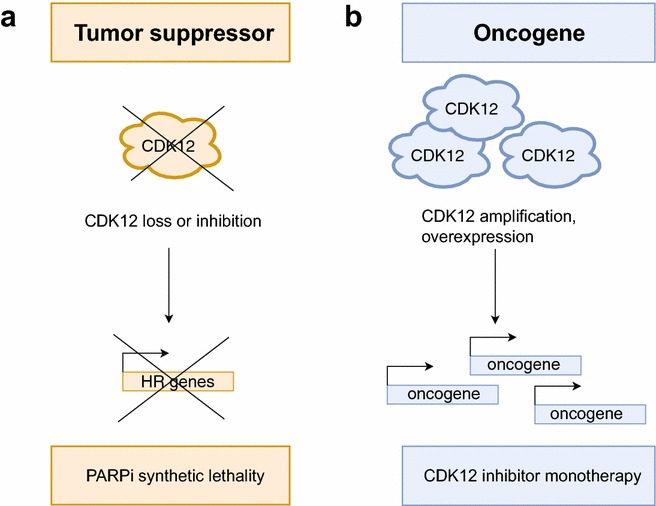
Role of CDK12 in cancer. **a** CDK12 has the tumor-suppressive properties. CDK12 loss-of function mutations lead to decreased expression of HR genes resulting in genomic instability and tumorigenesis. CDK12 loss or inhibition sensitizes tumor cells to PARP1/2 inhibitors. **b** CDK12 has oncogenic properties. CDK12 amplification might lead to increased expression of various oncogenes and consequently participate in tumorigenesis. Therefore targeting CDK12 with specific inhibitors in these tumors could be beneficiary for patient treatment

Taken together, the dual role of CDK12 in cancerogenesis could be explained by the fact that CDK12 is essential for expression of both tumor suppressors and oncogenes, and it participates in multiple cellular processes. In different tissues and cell types, the process of tumorigenesis depends on amplification of particular oncogenes or loss of suppressors. Consequently, CDK12 can act as a tumor suppressor or its amplification can contribute to cancerogenesis depending on cellular context (Fig. 1).

## Conclusions

CDK12 is a transcription-associated CDK essential for multiple cellular processes, including splicing, differentiation and DDR. Despite the fact that CDK12 has been extensively studied, our understanding of its functions remains limited. In vitro models will be instrumental to identify CDK12-associating factors and additional kinase targets and to elucidate whether it is a general transcriptional regulator or a specific factor for particular sets of genes. Mouse models recapitulating CDK12 loss or gain of function will be illustrative in studying particular aspects of diseases and development. Elucidation of CDK12 functions would lead to better assessment of its roles during tumorigenesis.

CDK12 mutations and amplification in tumors have been documented in an increasing number of studies. Recently developed CDK12 inhibitors constitute not only powerful research tools but also promising anti-cancer drugs. CDK12 inhibitor monotherapy could be useful for cancer patients tumors with overexpressed and activated CDK12. CDK12 inhibition increases sensitivity of cells to PARP1/2 inhibitors, thus presenting a potential strategy for targeting PARP1/2-resistant tumors.

## Abbreviations

cdk: cyclin-dependent kinase
CTD: C-terminal domain
RNA pol II: RNA polymerase II
DDR: DNA damage response
CrkRS: Cdc2-related kinase with an arginine/serine-rich (RS) domain
HR: homologous recombination
P-TEFb: positive transcription elongation factor b
Paf1: pol II associated factor 1
Nrf: nuclear respiratory factor
CstF77: cleavage stimulation factor 77 kDa subunit
CstF64: cleavage stimulating 34 factor 64
SC35: SR splicing factor 2
SFRS1: serine/arginine splicing factor1
c-MYC: MYC proto-oncogene
HGSOC: high-grade serous ovarian cancer
PARP: poly (ADP-ribose) polymerase
TNBC: triple-negative breast cancer
ER: estrogen receptor
HER-2: human epidermal growth factor receptor 2
